# The National Insurance Medical Association

**Published:** 1912-01-06

**Authors:** 


The Hospital

The Workers' Newspaper of Administrative Medicine and Institutional Life, Administration, National Insurance and Health.

No. 1330, Vol. LI. SATURDAY, JANUARY 6th, 1912. PRICE ONE PENNY.

### THE NATIONAL INSURANCE MEDICAL ASSOCIATION.

Under the above title an attempt is being made from headquarters in Edinburgh to disunite and divide the profession on the subject of the Insurance Act. The promoter, or at any rate the ostensible organiser, of this attempt is Dr. C. F. Knight, an Irish graduate of thirty-five years' standing who teaches anatomy in Edinburgh. He is collecting the names of those who will be willing to accept work under the Insurance Act, no matter whether the "six points" of the British Medical Association's policy be conceded or not. His utterances and his propaganda are receiving, not unnaturally, advertisement and encouragement in those organs of the governing party which are doing their utmost to belittle the efforts of the profession to escape from the appalling tyranny and injustice created for its members through the inefficiency (or worse) of the Council of the British Medical Association by the Insurance Act as it stands to-day upon the Statute Book.

At the moment of writing no figures have been made public concerning the numbers of pledges received by Dr. Knight from the weak-kneed among the profession. It is true that announcements describing the progress of the canvass in glowing terms are published in the Radical press, *bien entendu*; but actual figures are not given, so that it is impossible to estimate the worth of these statements, and it is reasonable to discount them very freely. Meanwhile the arguments of the organiser of this movement demand a brief scrutiny. In *The Times* of December 30—possibly also elsewhere—he explains how, in his view, the Act is going to benefit, not only those of the profession who are foolish enough to join his Association now, but also the next generation of medical men.

First, he asserts that the outcry has arisen chiefly from the "hospital men" (probably he means visiting staffs of hospitals), who fear that the "insurance doctor" will deprive them of clinical material. No proof whatever of this is advanced; and no one who has attended the protest meetings held in various parts of the kingdom could make so baseless a statement. If there was anything perfectly certain, it was that those meetings were filled to overflowing by precisely those from whom the insured have hitherto obtained medical treatment—namely, the general practitioners of working-class districts.

He follows this by saying that the hospitals can make provision for the reception of patients willing to pay five shillings or seven and six weekly. To which it is only necessary to say that the Act gives the hospitals no power whatsoever to claim such sums out of the sick benefits conferred on the insured; and that even if it did, these sums constitute an eighth or less of the average cost of maintenance of the patient. So that seven-eighths of the income of the hospitals (in respect of insured persons) would still have to come from those sources of charity which the Act itself will do so much to dry up altogether.

Next Dr. Knight goes on to belaud the Insurance Act in its relations to medical education; that is, to the making of the modern practitioner. He says "it has been an acknowledged fact for years that practioners' . . . sons do not receive the training at the hospitals required in general practice. Here is the very thing they want; put the neonate into an insurance post—he gets the experience required, has a certain guaranteed income, and opportunities for working up a good private practice." Once more it must be noted that the argument, if such it can be called, is founded on a premiss which is quite incorrect. It is not "an acknowledged fact" that the hospitals fail to train young men for private practice. In so far as the kind of practitioner with whom Dr. Knight is apparently familiar does, all too commonly, fail to carry out properly and thoroughly the correct principles of medical and surgical practice which it is the aim of medical educators to instil, the statement is of some value as showing the bent of Dr. Knight's mind; but as a foundation for any serious deduction, it merely makes him ridiculous.

Passing over this fatal flaw, however, the second part of the paragraph, if more specious, is easily seen to be just as unsound. What sort of experience will the "neonate" (this is Dr. Knight's word, not ours) get when endeavouring to treat

120 patients a day in summer and 240 in winter? Surely just that habit of scamping work which the present club system of sweating the doctor has done so much to establish. What are the " opportunities " he is likely to have of " working up a good private practice "? Obviously, if they ever existed at all, employment of this degrading kind is the surest way of destroying them. Then, again, the " certain guaranteed income " dangled as the bait for the young practitioner. It has been proved to demonstration that this, under present arrangements, is not a " living wage " fairly commensurate to the work that will be exacted in return. This fact is, of course, the main foundation of the opposition of the medical profession to the Act; and no special pleading such as that of the National Insurance Medical Association's secretary will now avail to disguise it.

It will be a calamity indeed for the profession if there is any considerable percentage of men in it foolish enough to destroy the one remaining chance of escaping from a degrading slavery which is offered by a determined opposition to and boycott of the terms of medical service under the present Insurance Act. The outlook for the future of the medical profession is black enough in all conscience; but if the National Insurance Medical Association finds any root amongst members thereof, the death-knell of medicine as a learned profession and a progressive science will most certainly be very shortly tolled.

